# Humidity and temperature preference in two Neotropical species of sand flies

**DOI:** 10.1186/s13071-024-06325-2

**Published:** 2024-06-03

**Authors:** Rafael Vivero-Gomez, Daniela Duque-Granda, Jonathan A. Rader, Adam Stuckert, Ricardo Santander-Gualdron, Gloria Cadavid-Restrepo, Claudia X. Moreno-Herrera, Daniel R. Matute

**Affiliations:** 1 Grupo de Microdiversidad and Bioprospección, Facultad de Ciencias, Departamento de Biociencias, Laboratorio de Procesos Moleculares, Universidad Nacional de Colombia, Sede Medellín, Medellín, Colombia; 2 PECET (Programa de Estudio y Control de Enfermedades Tropicales), Universidad de Antioquia, SIU-Sede de Investigación Universitaria, Street 62 # 52-59Laboratory 632, 050003 Medellín, Postal Code Colombia; 3 Biology Department, University of North Carolina, Chapel Hill, USA; 4 Department of Biology and Biochemistry, University of Houston, Houston, TX USA

**Keywords:** Sand fly, *Lutzomyia*, *Pintomyia*, Climate change, Desiccation, Humidity preference, Temperature preference, Psychodidae, *Leishmania*, Leishmaniasis

## Abstract

**Background:**

Arthropods vector a multitude of human disease-causing organisms, and their geographic ranges are shifting rapidly in response to changing climatic conditions. This is, in turn, altering the landscape of disease risk for human populations that are brought into novel contact with the vectors and the diseases they carry. Sand flies in the genera *Lutzomyia* and *Pintomyia* are vectors of serious disease-causing agents such as *Leishmania* (the etiological agent of leishmaniasis) and may be expanding their range in the face of climate change. Understanding the climatic conditions that vector species both tolerate physiologically and prefer behaviorally is critical to predicting the direction and magnitude of range expansions and the resulting impacts on human health. Temperature and humidity are key factors that determine the geographic extent of many arthropods, including vector species.

**Methods:**

We characterized the habitat of two species of sand flies, *Lutzomyia longipalpis* and *Pintomyia evansi*. Additionally, we studied two behavioral factors of thermal fitness–thermal and humidity preference in two species of sand flies alongside a key aspect of physiological tolerance–desiccation resistance.

**Results:**

We found that *Lu. longipalpis* is found at cooler and drier conditions than *Pi. evansi*. Our results also show significant interspecific differences in both behavioral traits, with *Pi. evansi* preferring warmer, more humid conditions than *Lu. longipalpis*. Finally, we found that *Lu. longipalpis* shows greater tolerance to extreme low humidity, and that this is especially pronounced in males of the species.

**Conclusions:**

Taken together, our results suggest that temperature and humidity conditions are key aspects of the climatic niche of *Lutzomyia* and *Pintomyia* sand flies and underscore the value of integrative studies of climatic tolerance and preference in vector biology.

**Graphical Abstract:**

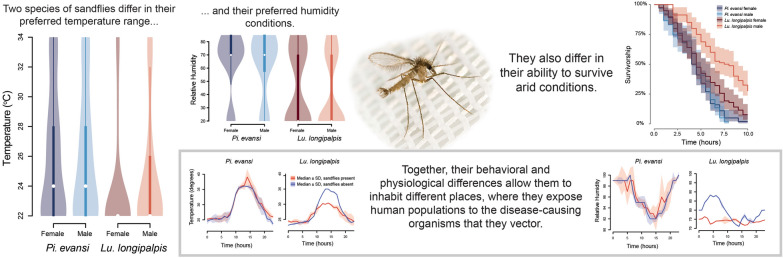

**Supplementary Information:**

The online version contains supplementary material available at 10.1186/s13071-024-06325-2.

## Background

Temperature and humidity are key environmental factors that determine the geographic distributions of species. As the mean global temperature increases, changes in rainfall and other climatic conditions will also change, and thus species will face substantially different regimes in temperature, humidity, and other climatic conditions. How species and communities will fare considering these changes is an important outstanding question. Some species are expected to go extinct while others are expected to migrate as their favored climatic conditions shift across the landscape. Some others will be able to remain in their original habitat and expand their geographic range [1–6]. The fate of each species in a changing world is largely, but not exclusively, dictated by the physiological and behavioral traits that determine their fitness. As a result, data on these traits are key to predicting species persistence and migration patterns in our changing climate.

In ectotherms, thermal fitness can be mediated by small variations in temperature and humidity [7–10]. Animals can make behavioral choices in response to changing environmental conditions to buffer themselves against unfavorable conditions (reviewed in [11]). Behavioral choices for favorable temperatures and humidity can mediate the microhabitats that animals inhabit [12]. More broadly, the physiological tolerance to and behavioral response for climatic conditions also defines a species’ overall geographic range [13–17].

Among the most important responses to predict are those of vector species, which transmit diseases to human populations because range overlap with vectors and shared habitat use influence the disease risk to which human populations are exposed. Arthropod-borne diseases are a particularly important group of diseases that are on the move, largely due to poleward migration of their vector species [18–20]. While much work has focused on identifying current and future movements of mosquitoes (e.g., [20–24]), other clinically relevant vector species have received less attention. To make predictions about future human disease risk from arthropod-borne pathogens, how the geographic range and particular habitat choices of vector species influence the disease risk to which human populations are exposed must be elucidated.

One group of vectors that is of particular importance are sand flies, which live in tropical and subtropical climates in both the New World and Old World tropics. The subfamily Phlebotominae comprises 1047 recognized species worldwide in 23 genera, with 554 species in the Neotropical region [25]. Many sand fly species are blood parasites, and at least 70 species pose a threat to human health by transmitting a number of pathogens that cause severe diseases, including leishmaniasis [26], bartonellosis [27, 28], and viral infections [29–32]. Leishmaniasis is a spectrum of diseases caused by around 20 species of the protozoan parasite *Leishmania*. Annually, more than 12 million people are infected with leishmaniasis and more than 2 million new cases are reported. In 2002, the number of recorded deaths due to *Leishmania* infection was around 60,000, a number that is, in all likelihood, a vast underestimate [33–35].

In the Neotropics, the main sand fly vector of leishmaniasis is the genus *Lutzomyia*, *sensu lato*, which has been recently been divided into several genera: *Lutzomyia, Pintomyia*, *Nissomyia*, *Psychodopygus*, and *Helcocyrtomyia*, among others [25, 36]. The group (henceforth referred to as Neotropical sand flies) is thought to be ecologically diverse, but specific ecological differences among species remain largely unstudied (cf. [37]). Because of their vector competency, Neotropical sand flies are of critical human health concern and these vectors have been hypothesized, on the basis of their current environmental preferences, to have the potential to expand their geographic range in response to climate change [38]. Indeed, recent cases of leishmaniasis have emerged among human patients in Texas who have not traveled outside of the USA [39–41]. These cases are strong evidence of a contemporary northward expansion of the disease and its vectors, or a change in the parasite reservoirs [42–44]. Additionally, veterinary cases have been recently observed in non-human mammals such as foxhounds in Virginia, and horses in Florida [45, 46]. Given the preponderance of evidence that these diseases are on the move, understanding the physiological and behavioral traits that foster range expansions in their vectors amid a changing climate is urgently needed.

A previous study used geographic occurrence to assess the ecological limits of the environmental niche of sand flies and indicated that several environmental correlates show evidence of strong phylogenetic signal and suggest that thermal fitness tends to be conserved among species of sand flies [47]. Sand flies and their relatives that originated in tropical lineages tend to remain tropical, which might explain the large species diversity of these vectors in the tropics. These comparative analyses reveal large-scale patterns in the evolutionary history of geographic range, but they cannot replace individual species assessments, which reveal the nuances of thermal niche.

There are at least three powerful reasons to explicitly study physiological and behavioral traits related to environmental conditions in vectors. First, thermal tolerance and preference are often genetically separable and thus need to be measured independently [48]. Occurrence records provide a good, but not perfect, correlation for environmental tolerance but do not capture behaviors that allow species to invade new places. Second, behavioral traits might reduce the range of environmental conditions experienced by an organism, buffering against selection on physiological traits [11, 49–51], and thus might either accelerate or hamper the possibility of invasion [11]. Finally, since many vector species are ectotherms, their behavioral syndromes also contribute to their association with humans because human settlements tend to be drier and warmer. Nonetheless, little is known about environmental preferences and tolerances in most vector species (*cf*. [52, 53]), and this is particularly true for sand flies.

In our study, we find that sand fly species differ in temperature and humidity preference, and in their humidity tolerance. We also find that there is a strong correlation between humidity preference and humidity resistance at the individual level in both species, suggesting a genetic correlation between the two traits. Our experiments further suggest that bacterial symbionts play a role in temperature preference. We suggest that behavioral traits need to be incorporated into the study of vector biology to better identify the precise thermal fitness components that could determine whether a species will expand its range in a changing world.

## Methods

### Specimen collection

We collected specimens in two locations that have yielded *Lu. longipalpis* and *Pi. evansi* collections in the past [54–56]. In the case of *Lu. longipalpis*, we set up three pairs of traps in the town of Ricaurte (Cundinamarca, Colombia). The traps in each pair were 20 m apart across a range of elevation between 333 m and 417 m. For *Pi. evansi*, we also set up three pairs of traps in the town of Colosó (Sucre, Colombia). These traps were all at sea level, as were the locations. For both locations, we measured the environmental conditions in each of the sampled sites using SensorPush Wireless Thermometer/Hygrometers (SensorPush, New York, USA) during a period of 3 days. While these measurements do not fully recapitulate the environmental variation of each site (for example, they do not encompass seasonal variation along the year), they do reveal the conditions at the time of sampling, and both species are short lived, with generation times of approximately 6–7 weeks. We compared the environmental conditions between sampling locations using linear models where either temperature or humidity were the response, and the trap was a fixed effect nested within the location (either Ricaurte or Colosó). Models were performed with the *stats* package using the *lm* function implemented in R (library *stats*, [57]). We followed the linear model with Tukey Honestly Significant Difference (HSD) post hoc pairwise comparisons (function *glht*, library *multcomp*, [58, 59]).

We used CDC-type light traps (2836BQX, BioQuip; Rancho Domingo, CA) to collect adults of the two species. Each trap was connected to a 6 V battery as a power source. The trap was operated for 24 h at a time. All the insects collected in the trap were put in a cooler with ice to immobilize the specimens. The whole collection was emptied into a Petri dish where the sand flies were selected and classified by species under a Leica dissecting scope. To identify sand fly species, we used two taxonomic keys [25, 36]. Individual sand flies were removed with tweezers and placed into 30 mL glass vials in sex-specific groups of up to 20 individuals for no more than 2 h before experimentation.

### Temperature preference

Previous experiments have used linear devices with a temperature gradient to study temperature choice (e.g., [60–63]). We sought to improve the device by generating a gradient stable over time. The device was composed of an aluminum sheet of 1000 × 160 × 5 mm shaped like a channel that allowed thermal conductivity (Additional file 1: Fig. S1). We placed three control modules at the ends of the channel: two at the cold end and one at the hot end. Each module on the cold side was composed of a fan, a Peltier plate, and a heat sink; the hot side module was composed of a single Peltier plate (Additional file 1: Figs. S1A and B). The aluminum channel was covered by an acrylic sheet that served as a lid and incorporated divisions that were manufactured to partially restrict the passage of insects, permitting greater control over them. We covered the inner part of the aluminum sheet with a muslin fabric to prevent insects from being affected by possible condensation on the inner chamber walls resulting from changing air temperature. To monitor the environmental conditions along the gradient, we placed ten pairs of digital temperature sensors outside the device (Additional file 1: Fig. S1) at 16.6 cm apart from each other and connected to a microcontroller to obtain real-time temperature readings using Arduino software. Additionally, we obtained relative humidity (RH) data using hygrometers (Hygro-Thermometer, BRIXCO, Model 5012C) placed within the channel. The temperature range of the thermocline was 22–34 ℃.

We evaluated the stability of the temperature gradient over time by measuring them in test trials without insects. To determine whether there was heterogeneity over time and among compartments in the device, we measured the temperature of the end compartments every 10 s for 30 min with no insects. We also studied whether sand flies showed a positional preference along the thermocline that was not related to differences in temperature. For females of the two species, we did assays with the thermocline off and assessed whether the positioning of the insects departed from a uniform distribution using a *χ*^*2*^ test (function *chisq. test*, library *stats*, [57]).

Once we validated the device with these two controls (see Results), we moved forward to measure the temperature preference in the two focal species of sand flies. The approach for the two species is identical. We collected specimens as described above (Specimen collection). Sex-specific groups of  ~ 50 individuals were anesthetized using ice for 2–4 min, a duration which allows for fast recovery. The anesthetized group was placed at the center of the pre-warmed thermocline. The insects were then allowed to explore the gradient for 60 min, at which point we recorded the position of each individual as a proxy for its climatic preferences. We ran two replicates with the males from each species and three replicates with females from each species. In total, we retrieved 170 *Lu. longipalpis* individuals (80 males and 90 females) and 242 *Pi. evansi* (91 males and 151 females). The lower number of males in our experiments reflects biases in capture rates.

To determine whether species and sexes differed in their temperature preference, we fit a linear mixed-effects model [64, 65] with temperature as the response, species and sex as the fixed effects, and an interaction term between the two effects. Replicate experimental runs were considered random effects. We conducted these analyses in R using the function *lme* (library *nlme*, [66]) followed by linear contrasts using the function *lsmeans* from the *lsmeans* library [67, 68].

### Treatment with tetracycline

Endosymbionts and other associated microbes have been shown to affect behavioral traits in animals [69, 70]. We studied whether associated bacteria were involved in temperature preference by exposing *Lu. longipalpis* females to tetracycline for 2 days. This treatment usually affects microbiome composition and diminishes the load of endosymbionts [71]. We collected 330 *Lu. longipalpis* females with an aspirator as described above (see specimen collection). We placed the group of females in a BugDorm cage and offered them a mixture of 10% sugar water supplemented with 50 uM tetracycline for 48 h. We removed the females from the cage, briefly cold-anesthetized them, and measured the temperature preference of tetracycline-exposed sand flies as described immediately above. We measured the preference of 268 treated *Lu. longipalpis* females and compared the temperature preference of treated and untreated females using a one-way analysis of variance (ANOVA; function *lm*, library *stats* [57]).

### Humidity preference

We measured the relative humidity (RH) preferences of *Lu. longipalpis* and *Pi. evansi* by monitoring the proportion of time during 45 min trials that sand flies spent in either a humid or dry portion of a 48-well polystyrene tissue culture plate (8 rows by 6 columns; Corning Incorporated, Life Sciences, Tewksbury, MA, USA). Sand flies were collected and maintained as described in the immediately previous section (Specimen collection), in groups of approximately 20 individuals (separated by sex).

To generate differences in RH, we filled the top three rows of a plate with super-saturated KH_2_PO_4_ solution, the next three with NaCl, and the bottom three rows with super-saturated LiCl. The headspaces of these three salts differ by their hygroscopic properties and they generate RHs of  ~ 85%, 70%, and 25%, respectively, in the headspace above the rows. We then covered the top of each plate containing the salt solutions with 300 micron nylon netting (MegaView Science Co., Ltd. Taichung, Taiwan) and placed a 3-D printed plastic frame on top of the netting. This plastic frame was partitioned into six columns, with each column as wide as one column of the 48-well plate and covering three wells containing the humid-generating solution and three containing the dry-generating solution (see a similar design in [72, 73]; .stl file for 3-D printing available in Additional file 2).

To transfer sand flies into the device, we cold-immobilized groups of seven flies (separated by sex and species) by placing them in a Petri dish in a Styrofoam chest with ice for ~ 5–10 min. We then transferred one group of flies into each of the chambers formed by the plastic frame over each plate for a total of ~ 20 sand flies per replicate. The frame was then covered with 3 mm glass, leaving the flies with ~ 5 mm to move around on top of the plate. Each tray was placed at 26 °C, and the insects were allowed to recover from cold knock-down and sort themselves across the plate for 30 min. We recorded the position of the sand flies every 10 min. We ran between 3 and 12 replicates per genotype (species × sex) with ~ 20 sand flies per replicate. In total, we observed 486 sand flies for this portion of the research. We fit a linear model in which the humidity preference was the response, and species and sex were the two fixed effects to determine whether there were differences in humidity preference. We used the R function *lm* (library *stats*, [57]). The model also included the interaction between these two terms. We used a post hoc test to determine whether there were differences between species (function *glht*, library *multcomp*, [57]).

### Desiccation resistance

We measured how long sand flies of the two species could survive in extreme desiccation conditions. Desiccation resistance was measured by placing 20 females or males in 30 ml empty vials, which in turn were placed in a glass desiccator with 200 g of Drierite and kept at 21 ℃ [74, 75]. The relative humidity was kept under 20% and was measured with a hygrometer. Flies were checked every 30 min and the time of death recorded for each sand fly. We ran between three and five experimental batches per genotype. In total, we measured the trait for 158 *Lu. longipalpis* individuals (78 females and 80 males), and 159 *Pi. evansi* (100 females and 59 males). To analyze whether there were differences among genotypes, we used a survival analysis and a Cox regression (function *cph*, library *rms*, [76]). To determine the significance of the effects, we compared linear models that included and excluded the effect to be tested using a likelihood ratio test (LRT; function *lrtest*, library *lmtest*, [77]). To visualize the results, we generated plots with the *‘survplot’* function.

We did a second experiment to quantify whether desiccation resistance and humidity preference were correlated at the individual level. We measured humidity preference using the same arenas described above but conducted the experiment with individual sand flies rather than in groups. We used the relative proportion of time spent in each area of the arena as a proxy of humidity preference. We measured 60 individuals per species (30 per sex). After 30 min, we removed the flies from the arena using a mouth aspirator (1135A Aspirator–BioQuip; Rancho Domingo, CA). We then transferred each fly to an individual 30 mL vial and measured their desiccation resistance in the same way as described immediately above. At the end of the experiment, we kept all individuals in ethanol to measure their individual size (described immediately below). We calculated the correlation between these two individual phenotypes using the R function *cor. test* (library *stats*, [57]) for each of the two species. We generated distributions of the two correlation coefficients using 1000 bootstrapped values (function *boot*, library *boot*, [78, 79]) and compared them using a Wilcoxon rank sum test with continuity correction (function *wilcox. test*, library stats, [57]). Because body size can be a strong predictor of desiccation resistance, we also calculated the correlation between desiccation resistance and thorax length (see immediately below) using the same protocol.

### Body size

Resistance to desiccation is correlated with body size in some terrestrial arthropods, including *Drosophila* [80, 81], so we investigated the relationship between body size and desiccation resistance in Neotropical sand flies. We used thorax length as a proxy of body size. We used a Leica M80 Stereo Zoom Microscope dissecting scope for all imaging. To measure the length of the thorax, each sample was placed in a 0.01 mm stage micrometer (Amscope MR095) and we recorded the distance between the sternum and the notum. We measured ~ 20 individuals (between 19 and 21) per species and sex for a total of 80 individuals (2 sexes × 2 species). To determine whether there was heterogeneity in the size, we used a factorial linear model with thorax length as the response, and species and sex as the effects. To compare the sizes of the four genotypes, we used a Tukey post hoc test (function *glht*, library *multcomp*, [59, 82]).

## Results

### Environmental conditions

**Fig. 1:**
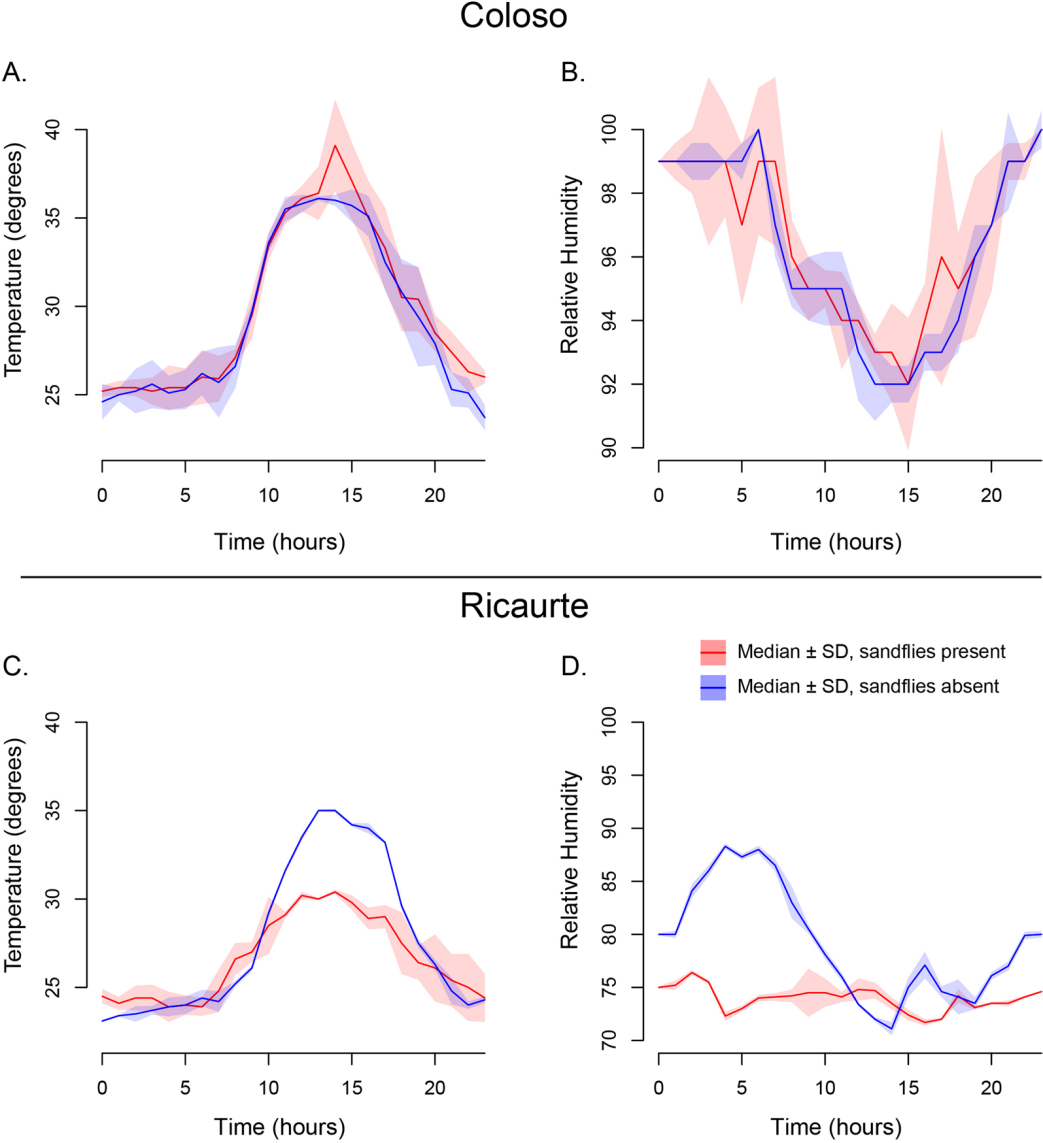
Environmental conditions during the collection period in two sampling locations. The trend lines depict median temperature and humidity conditions during the collection period at both localities; polygons around the trend line show variation among specific trap sites at each location. **A** Temperature in Colosó. **B** Relative humidity (RH) in Colosó. **C** Temperature in Ricaurte. **D** RH in Ricaurte. Red: traps that yielded sand fly specimens. Blue: traps that yielded no specimens

We collected *Lu. longipalpis* in the highlands (Ricaurte locality) and *Pi. evansi* in the lowlands (Colosó locality) of Colombia. We measured the temperature and humidity levels of the environments in which the species were present and locations in which they were absent. Figure 1 shows the environmental conditions during the sampling period. Colosó, where *Pi. evansi* was collected, was warmer than Ricaurte, where we collected *Lu. longipalpis*, as expected by their altitudinal difference, (°*T*_Mean-Colosó_ = 29.396 °C, °*T*_Mean-Ricaurte_ = 27.077 ℃; LM: ANOVA, *F*_1280_ = 25.458, *P* < 0.0001). Similarly, Colosó was also more humid than Ricaurte (RH_Mean-Colosó_ = 96.403%, RH_Mean-Ricaurte_ = 76.576%; LM: *F*_1280_ = 2270.757, *P* < 0.0001). We fitted linear models (LM) for each of the two locations to determine whether there was microspatial heterogeneity within sampling locations. In Ricaurte, traps where *Lu. longipalpis* were collected showed a slightly lower mean temperature and higher humidity than locations where *Lu. longipalpis* was not present (Table 1; temperature LM: ANOVA, *F*_1140_ = 4.097, *P* = 0.045; humidity LM: ANOVA, *F*_1140_ = 111.425, *P* < 0.0001). This difference is notable because the traps were placed within 20 m of each other and highlight the existence of microhabitat differences within a location. Furthermore, traps with or without *Pi. evansi* in Colosó showed no difference in their environmental conditions (Table 1; temperature LM: ANOVA, *F*_1140_ = 0.138, *P* = 0.711, humidity LM: ANOVA, *F*_1140_ = 0.095, *P* = 0.758). These results pose the possibility that at least some species of sand flies are cuing in on subtle climatic differences to choose their preferred habitat, a hypothesis we explored in controlled experiments as follows.

**Table 1:** Environmental conditions in the two localities sampled in this study

	Ricaurte	Humidity	Colosó	Humidity
	Temperature		Temperature	
With sand flies	26.522	73.903	29.532	96.472
No sand	27.632	79.249	29.260	96.333

All calculations are based on 3 points, for a total of 12 points

### Temperature preference

Temperature preference has been determined to be an important component of habitat choice in dipterans (e.g., [61, 73]). Since we were using a newly designed device, we studied the stability of the environmental gradient. Additional file 1: Fig. S1C shows the results for these assessments, which revealed that the device takes ~ 25 min to stabilize. These results suggest that after that warm-up period, the gradient remains stable. A second control was to determine whether sand flies from the two different species show positional differences along the gradients not related to temperature. When we allowed sand flies to distribute themselves along the device when it was off, we found no deviations from a uniform distribution in either of the species (*Lu. longipalpis*: *χ*^2^ = 7.487, *df* = 6, *P* = 0.278, *Pi. evansi*: *χ*^2^ = 7.6, *df* = 6, *P* = 0.269). These two experiments indicate that our thermocline is a functional tool to measure temperature preference in sand flies and we thus moved forward with insect preference experiments.

**Fig. 2:**
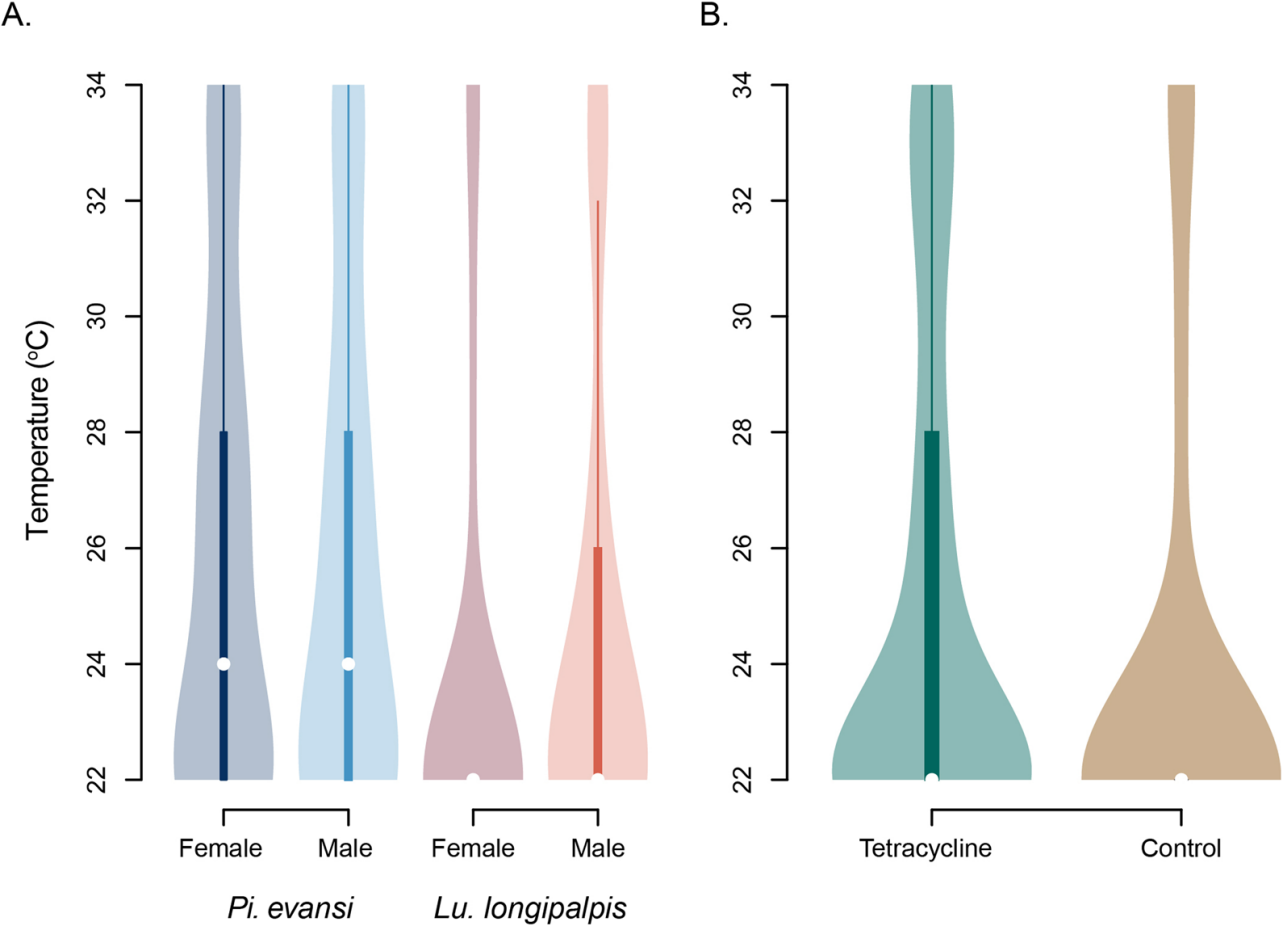
Temperature preference variation in sand flies. **A** *Lutzomyia longipalpis* and *Pintomyia evansi* differ in their temperature preference. **B** Tetracycline affects the temperature preference in *Lu. longipalpis* females. Note that the distribution of temperature preference in *Lu. longipalpis* females is shown in both panels

We measured the temperature choice in the two species of sand flies, separating by sex to avoid potential effects of courtship behavior or mating. Figure 2A shows the mean temperature preference for both species and both sexes. A linear mixed-effects model revealed that the two sand fly species differed in their temperature preference (*F*_1406_ = 18.011, *P* < 0.0001) with *Lu. longipalpis* having a lower temperature preference (mean = 24.200, *sd* = 4.039) than *Pi. evansi* (mean = 25.926, *sd* = 4.290). The same linear model revealed that sexes did not differ in their temperature preference (*F*_1406_ = 0.015, *P* = 0.902) and that there was no significant interaction between species and sex (*F*_1406_ = 1.216, *P* = 0.271). These results suggest that there is heterogeneity in temperature preference between species of sand flies.

We explored whether bacterial symbionts affected temperature choice in *Lu. longipalpis* females. We found that *Lu. longipalpis* females exposed to the antibiotic show a significantly higher temperature preference than females not exposed to the treatment (Fig. 2B; LM, *F*_1356_ = 4.724, *P* = 0.030; mean_treated_ = 24.948, *sd*_treated_ = 4.234; mean_untreated_ = 23.844, *sd*_untreated_ = 3.957). These results suggest that at least in some species of sand flies, bacterial symbionts might have an effect in behavioral traits. Please note that we did not directly measure the impact of the tetracycline treatment on the microbiome or study the effect of tetracycline on temperature choice in *Pi. evansi* or in *Lu. longipalpis* males.

### Humidity preference

**Fig. 3:**
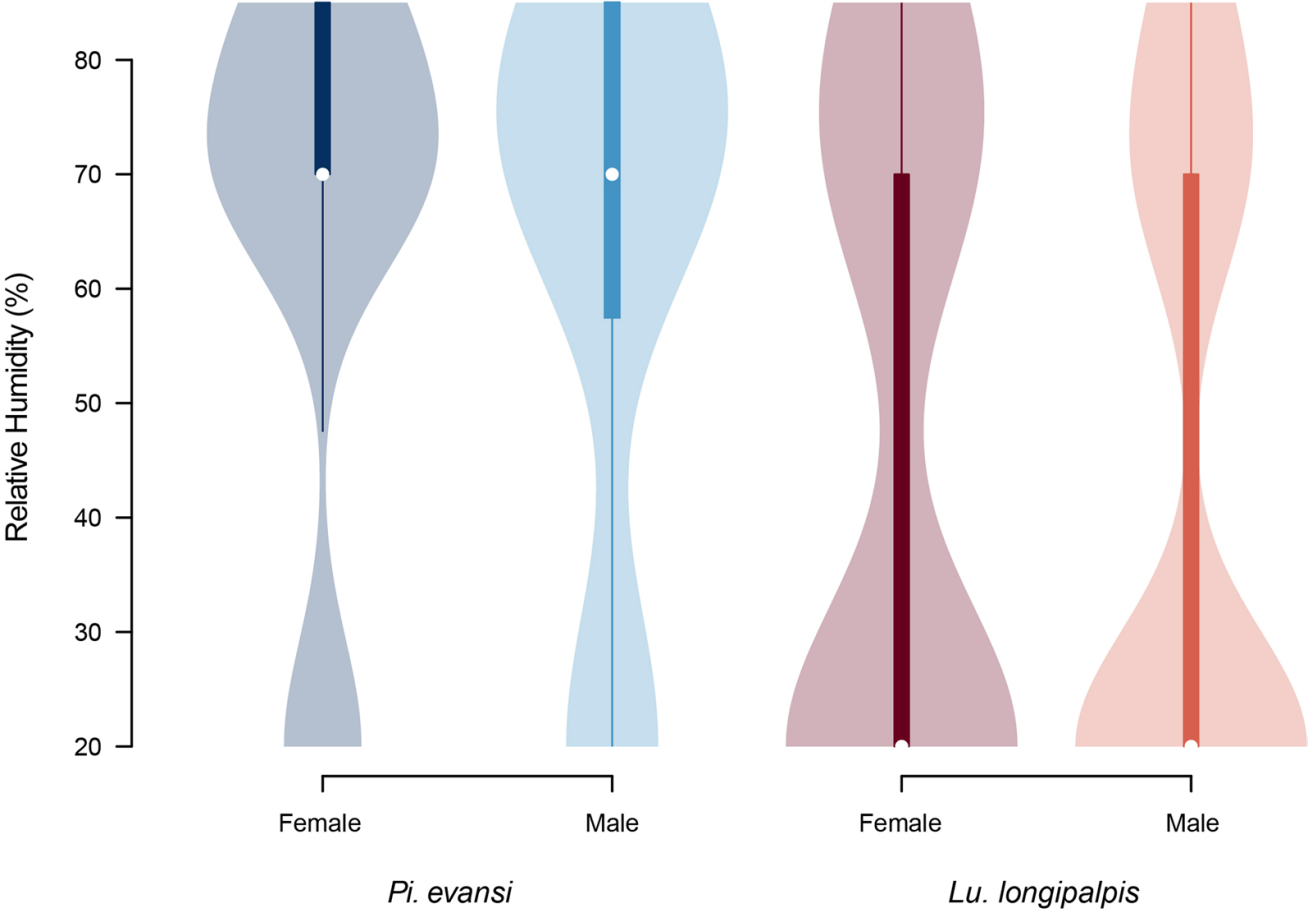
Humidity preference differs between the two species of sand fly. *Lutzomyia longipalpis* and *Pi. evansi* differ in their RH preference in laboratory experiments. Sex was not a significant factor within species

An environmental correlate of temperature is environmental humidity. We studied the extent to which the two focal species of sand flies preferred different humidity conditions using a controlled lab setting with a humidity gradient ranging from 20% to 85% humidity. Figure 3 shows the humidity levels preferred by each of the four genotypes included in this study. A linear mixed model revealed a similar pattern to that of temperature, in which the species identity played a strong effect on humidity preference (*F*_1482_ = 70.393, *P* < 0.0001), but neither sex nor the species × sex interaction was significant (sex: *F*_1482_ = 1.271, *P* = 0.260; sex × interaction: *F*_1482_ = 0.031, *P* = 0.861). Of the two species, *Lu. longipalpis* preferred dryer conditions (mean = 43.339, *sd* = 27.904) than *Pi. evansi* (mean = 63.989, *sd* = 23.838), a difference that was significant according to Tukey HSD post hoc tests (|*t*|= 8.396, *P* < 0.0001). These results suggest that, besides temperature preference, humidity preference also contributes to habitat choice in these two species of sand flies.

### Desiccation resistance

**Fig. 4:**
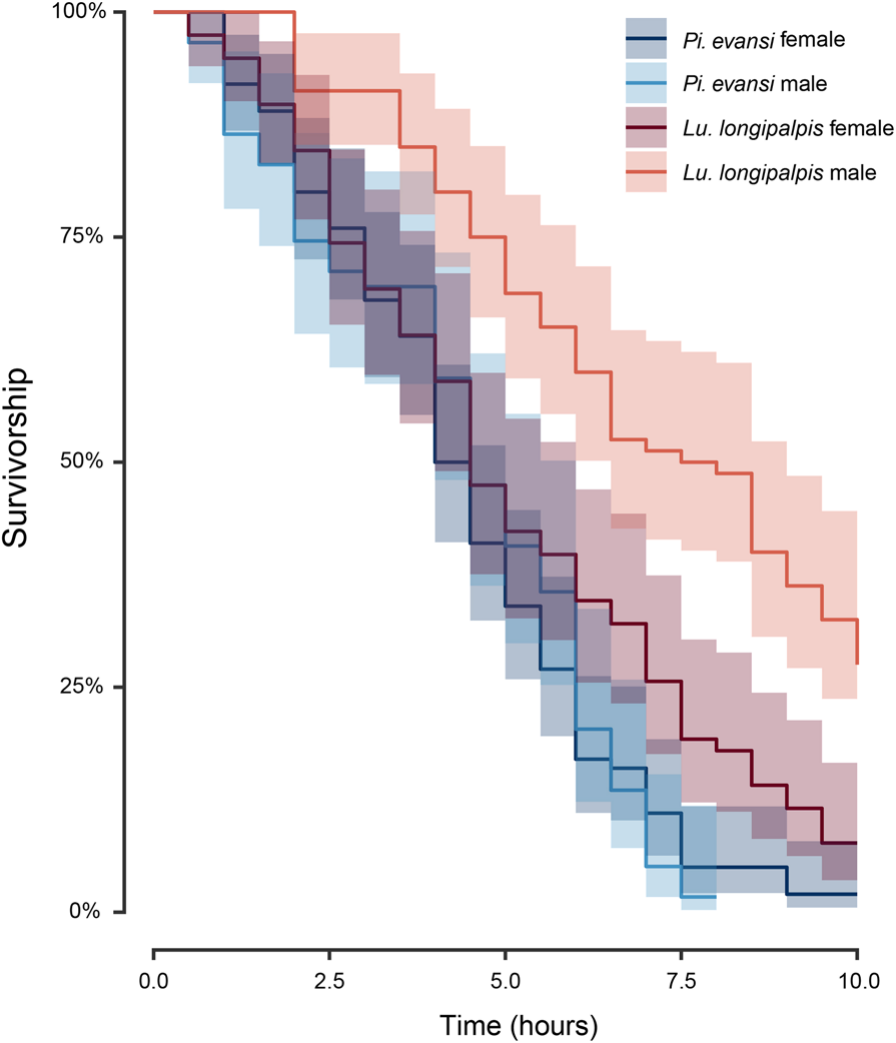
Survival plots of two species of sand flies in extreme desiccation conditions. All experiments were conducted for 10 h, or until all individuals had died. We ran experiments for at least three replicates per genotype. The two species of sand flies differ in their physiological tolerance to desiccating conditions, and within *Lu. longipalpis*, males were especially resistant to desiccation

We measured a third, non-behavioral trait, desiccation resistance, by measuring how long individuals from the two species survived extreme desiccation conditions. Figure 4 shows the survival curves of the four genotypes and Table 2 shows the mean time to death in extreme desiccating conditions. We found significant differences between species (LRT, *χ*^*2*^ = 17.325, *df*= 1, *P* < 0.0001), and sexes (*χ*^*2*^ = 17.922, *df* = 1, *P* < 0.0001). The interaction between species and sex was also significant (LRT, *χ*^*2*^ = 21.670, *df* = 1, *P* < 0.0001). *Lutzomyia longipalpis* males survive desiccation the longest of the four genotypes, followed by *Lu. longipalpis* females, which survive longer than *Pi. evansi* from either sex. Males and females from *Pi. evansi* show equivalent survival (Table 2).

**Table 2:** Pairwise comparison between the desiccation resistance of the four genotypes in this study

			Tukey tests			
Genotype	Mean	SD	*Lu. longipalpis* females	*Lu. longipalpis* males	*Pi. evansi* females	*Pi. evansi* males
*Lu. longipalpis* females	5.128	2.759	*	< 0.001	0.045	0.076
*Lu. longipalpis* males	7.044	2.780	4.376	*	< 0.001	< 0.001
*Pi. evansi* females	4.420	2.165	2.603	6.871	*	0.999
*Pi. evansi* males	4.373	2.147	2.399	6.196	0.140	*

Body size is an important predictor of desiccation resistance in arthropods [80, 83]. We therefore studied whether there was a size difference between the two species or sexes that might in turn explain variation in humidity preference or desiccation resistance. We found that species (*F*_1,76_ = 24.445, *P* < 0.0001) and sex (*F*_1,76_ = 30.783, *P* < 0.0001) both have an effect on body size. Post hoc tests revealed that *Lu. longipalpis* is larger than *Pi. evansi* (Tukey HSD, |*t*|= 4.360, *P* < 0.0001), and that there is sexual dimorphism in which females are larger than males (Tukey HSD, |*t*|= 2.964, *P* = 0.004). Table 3 presents all the pairwise comparisons among genotypes. Notably, the largest genotype, *Lu. longipalpis* females, is not the one with the highest level of desiccation resistance, indicating some other physiological syndrome leading to higher resistance. Overall, our results suggest that *Lu. longipalpis* survives better in desiccating conditions, that it also prefers drier environments, and that differences among genotypes are not completely explained by body size differences.

**Table 3:** Thorax length suggests body size differences among sand fly species and sexes

			Tukey tests			
Genotype	Mean (mm)	SD (mm)	*Lu. longipalpis* females	*Lu. longipalpis* males	*Pi. evansi* females	*Pi. evansi* males
*Lu. longipalpis* females	0.053	2.455 × 10^–3^	*	< 0.001	< 0.001	< 0.001
*Lu. longipalpis* males	0.048	2.705 × 10^–3^	4.855	*	0.981	0.045
*Pi. evansi* females	0.049	3.670 × 10^–3^	4.360	0.380	*	0.021
*Pi. evansi* males	0.046	2.573 × 10^–3^	7.419	2.654	2.964	*

We measured the thorax (notum to sternum) of individual sand flies as a proxy of body size

**Fig. 5:**
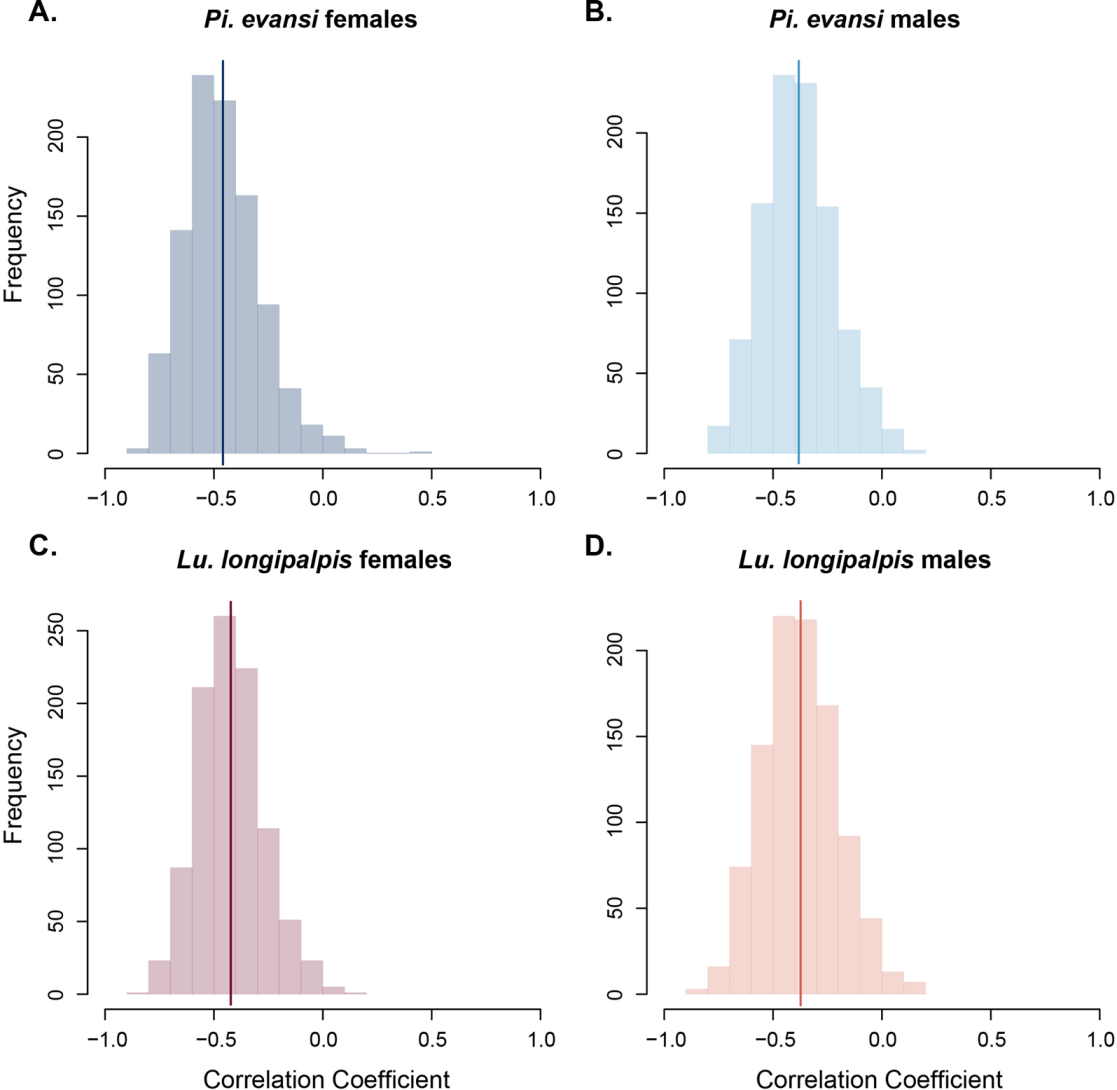
Sand flies that prefer more humid temperatures are more sensitive to desiccating conditions. The two species show intraspecific phenotypic variation in desiccation resistance and humidity preference that is negatively correlated (i.e., individuals that prefer more humid conditions are more likely to die early in desiccating conditions). Histograms show Pearson’s correlation tests of 1000 bootstrapped samples. **A** *Pi. evansi* females. **B** *Pi. evansi* males. **C** *Lu. longipalpis* females. **D** *Lu. longipalpis* males

Finally, we studied whether the variation in humidity preference and desiccation resistance within species were correlated by measuring the two traits in the same individuals. Notably, desiccation resistance was not correlated with body size within any of the four genotypes (Tukey HSD, |*t*| < 1.552, *df* = 28, *P* < 0.132 in all four cases). In general, individuals with higher desiccation resistance also showed a preference for higher humidity in both species, *Lu. longipalpis* and *Pi. evansi*. Correlation between these two traits was significant in both species and sexes (Table 4) but the magnitude of the correlation differed between the two species. Figure 5 shows the distribution of the bootstrapped coefficients for each of the four genotypes. All pairwise comparisons were significantly different (Table 4). This result suggests that there is phenotypic variance, not related to body size, in the traits that confer desiccation resistance in both species, and that these traits tend to be correlated but that the extent of correlation varies among species.

**Table 4:** Correlation between individual humidity preference and desiccation resistance for four genotypes of sand flies

				Wilcoxon test			
Genotype	Spearman’s *Rho*	Confidence interval	*P*-value	*Lu. longipalpis* females	*Lu. longipalpis* males	*Pi. evansi* females	*Pi. evansi* males
*Lu. longipalpis* females	−0.428	[−0.683, −0.080]	0.01834	*	< 0.001	< 0.001	< 0.001
*Lu. longipalpis* males	−0.372	[−0.646, −0.014]	0.043	398,769	*	< 0.001	0.025
*Pi. evansi* females	−0.479	[−0.716, −0.143]	0.008	415,513	328,246	*	< 0.001
*Pi. evansi* males	−0.391	[−0.659, −0.036]	0.032	570,769	471,100	353,615	*

Pairwise comparisons between correlation coefficients were done using a Wilcoxon test on bootstrapped distributions for each coefficient (*n* = 999)

## Discussion

In this report, we present evidence of interspecific differences in behaviors associated with thermal fitness among species of Neotropical sand flies, the vectors of the etiological agents of leishmaniasis and other serious diseases. Behavioral and physiological experiments are necessary to understand the habitat choice and tolerance to environmental conditions of individual species. These experiments supplement inferences of geographic range and climatic tolerance from occurrence data. Lab assessments reveal the physiological limits of species occurrence; suitability analyses reveal the realized range that can be affected by abiotic factors and by biotic interactions with competitors, predators, and hosts. While we focused on two of the Neotropical species of sand flies, our report serves as a blueprint to systematically characterize the behavioral and physiological components of climatic fitness in other disease vectors.

Our results allow us to separate thermal fitness into at least two behavioral components and one physiological component: temperature preference, humidity preference, and desiccation resistance. Notably, individuals that showed preference for more humid conditions tended to be more susceptible to extreme desiccation conditions (i.e., died faster). This pattern suggests the potential for a genetic correlation between humidity preference and desiccation resistance in both species of sand flies. Further studies will address whether this phenotypic variation in both traits is caused by the same alleles or whether they are genetically separable components of thermal fitness. These, of course, are not the only traits that affect thermal fitness, and endurance at high and low temperatures is another critical component that warrants additional attention.

Our results also reveal other facets of habitat choice in sand flies. We find that treating *Lu. longipalpis* with tetracycline affects their temperature preference. This effect can be explained by an involvement of bacterial endosymbionts, of other bacterial communities, or of mitochondria in temperature choice. *Wolbachia* (*Wb*), for example, is one of the best characterized bacteria in dipterans, and experiments in *Drosophila* have conclusively demonstrated that *Wb* can affect thermal preference [70]. Polymerase chain reaction (PCR) screens have revealed that *Wolbachia* is not only present, but in some cases highly prevalent, among species of sand flies [84]. In Brazil, 26.3% of *Lu. longipalpis* specimens were positive for *Wb*, with a large range across municipalities (8.4–60.0%). *Wolbachia* infections are also present in *Pi. evansi* (which we did not treat with tetracycline) but at a much lower rate (~ 2% of individuals [54]). A second possibility is that our tetracycline treatment disrupts mitochondrial stoichiometry and metabolism. In *Drosophila*, tetracycline can cause a significant increase in mtDNA density in naturally *Wolbachia*-uninfected but not in naturally *Wolbachia*-infected lines [85]. Our results serve as the first suggestion that antibiotics can influence habitat choice in disease vectors, but our current experiments do not allow us to identify the source of the effect.

Our experiments have caveats that are worth mentioning. First, all our experiments used individuals collected in the wild, which did not allow us to control for all factors known to affect temperature (reviewed in [86]. In insects, for example, age and mating status have an effect on thermal fitness (e.g., [87]) but the magnitude of the behavioral preference change is not sufficient to override interspecific differences. This collection scheme also limits our ability to interpret the effect of tetracycline because we did not screen individuals for the presence of *Wb* or other endosymbionts. Though some preliminary surveys have characterized the microbiome of *Lu. longipalpis* [88, 89] reviewed in [90]) and of *Pi. evansi* [55], the study of the microbial community in *Lutzomyia* remains in its infancy. Nonetheless, it has become apparent that the microbiome can influence multiple traits, including some that are related to vector competency [88]. The applied tetracycline in our experiments could have affected *Wb* titers, gut microbiome, or host physiology. A proper characterization of the associated microbes in the experimental populations, along with a characterization of the mitochondrial metabolism, will be required before determining the precise mechanism of this behavioral change, and more broadly to understand how the microbiomes of vectors influence the risk they pose to human health.

Tropical vector species are expected to increase their ranges as global warming proceeds [22, 24, 38, 91, 92]. Indeed, vector species represent some of the most spectacular cases of biological invasion. *Aedes aegypti*, for example, has increased its range from Africa across the world in the last six centuries following patterns of human movement [22, 93, 94]. A second species, *Aedes albopictus*, has shown an explosive increase in geographic range in just the last few decades. Both species are expected to further increase their ranges on the order of hundreds of kilometers per year [22, 24]. In the case of sand flies, multiple species have been projected to expand their range if the global temperature continues warming [38]. The expansion may not be solely latitudinal for all species, though. *Lutzomyia longipalpis*, for example, might expand its range within the tropics and subtropics but not into the temperate zones [92]. The Caribbean islands and southern Florida currently harbor habitats that might be prone to successful colonization by *Lu. longipalpis*, even though no records of the species exist for these areas. Southern Brazil, the Orinoco region of Colombia, and the Pacific coast of Ecuador and Peru may also provide suitable habitats for the species. These models, of course, are not deterministic and only reveal the potential for invasion.

## Conclusions

Understanding the thermal niche of vector species has clear implications for understanding future disease risk. According to the World Health Organization (WHO, 2022), climate change is a key driver of the rising number of leishmaniasis cases around the world. Even small variations in temperature can affect the development of pathogens and parasitic organisms such as *Leishmania*, leading to their transmission in areas where the disease was not previously present. Additionally, changes in exposure to insect vectors resulting from human movement, changes in land use, and shifting geographic distributions of insect populations following temperature, humidity, and rainfall fluctuations highlight the importance of integrative studies of climatic tolerance and preference in insect vectors.

### Supplementary Information

Supplementary Material 1.

Supplementary Material 2.

## Data Availability

All data and analytical code are available at https://figshare.com/articles/dataset/Humidity_and_temperature_preference_in_sand_flies/25813558
