# Supplementary material for: Humidity and temperature preference in two Neotropical species of sand flies
Source: Parasit Vectors. 2024 Jun 3;17:246. doi: 10.1186/s13071-024-06325-2 (PMC11149334; doi:10.1186/s13071-024-06325-2)

**ADDITIONAL FILE 1: FIGURES S1-S3**

**Humidity and temperature preference in two Neotropical species of sandflies**

Rafael Vivero-Gomez, Daniela Duque-Granda, Jonathan A. Rader, Adam Stuckert, Ricardo Santander-Gualdron, Gloria Cadavid-Restrepo, Claudia X. Moreno-Herrera and Daniel R. Matute

**FIGURE S1. Thermocline design used in this report. A.** Diagram of the electrical circuit. P1 and P2 represent the Peltier plates located on the cold side. P3 represents the Peltier plate located on the hot side. V1 and V2 represent the fans of the cold side modules. The ten (10) temperature sensors are drawn at the bottom of the image represented with the letter "M". The other components correspond to current transformers (AC/DC and DC/DC), a relay that controls the voltage on the Peltier board on the hot side, an Arduino microcontroller, and an OLED display. **B.** A picture of the thermocline device. 1: Control box; 2: Current transformer; 3: Fans at the cold end of the device; 4: Aluminum channel, isolated with expanded polystyrene, divisions, and lid in acrylic; 5: Hot end. **C.** Temperature changes in the cold and hot side of the thermocline over a ~30 min period without insects. Circles: hot side. Triangles: cold side.


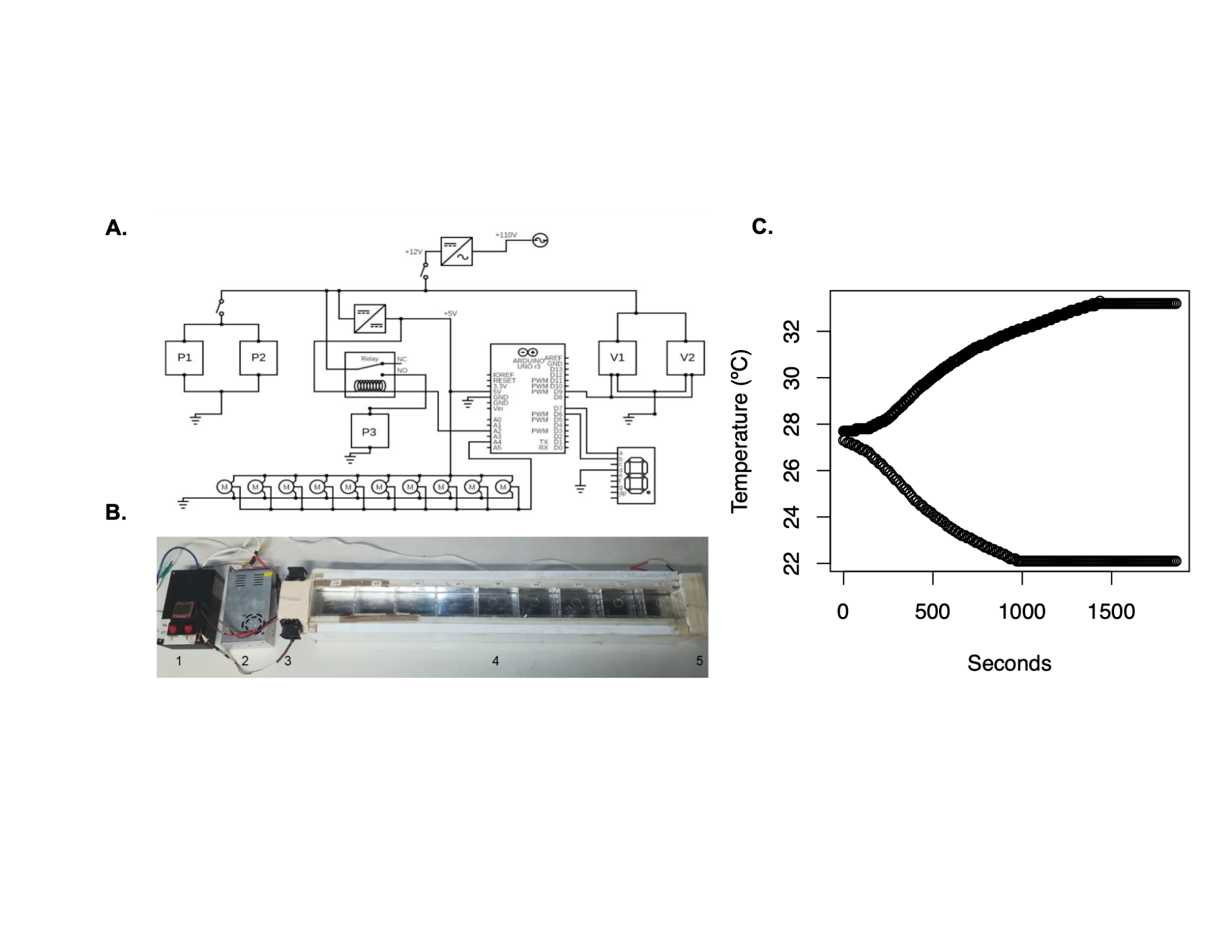


**FIGURE S2. Individual data points showing environmental conditions during the collection period in two sampling locations.** **A.** Temperature in Colosó. **B.** Relative humidity (RH) in Colosó. **C.** Temperature in Ricaurte. **D.** RH in Ricaurte. Red: traps that yielded sandfly specimens. Blue: traps that yielded no specimens.

**
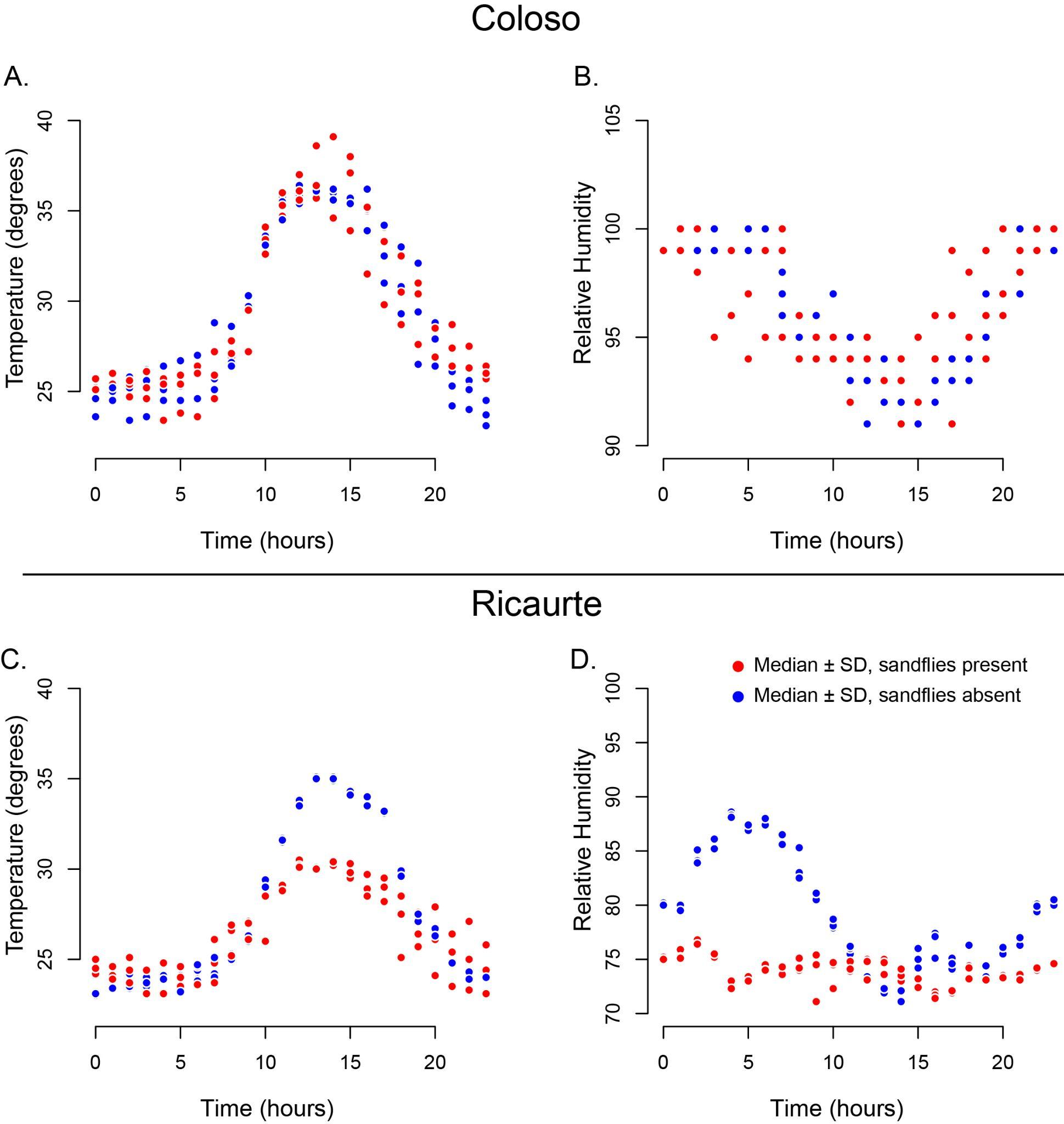
**

**FIGURE S3. Body size differs between species and sexes in sandflies. We measured between 19 and 21 individuals per genotype.** As a proxy of body size we used thorax length measured as the distance between the sternum and the notum.


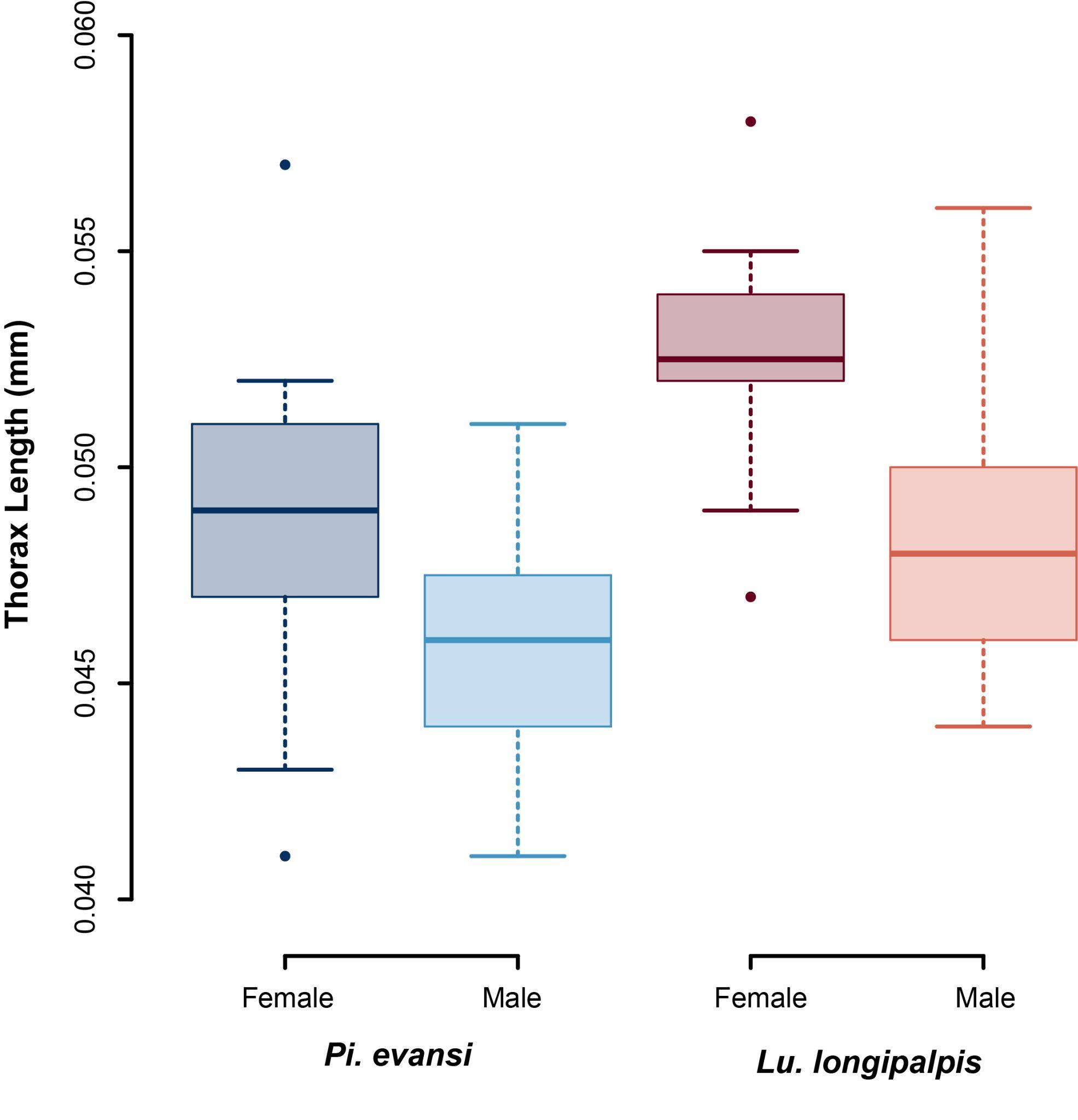

Supplement: Supplementary file 1 — Supplementary Material 1. [file 13071_2024_6325_MOESM1_ESM.docx]
